# High-Performance 3D Compressive Sensing MRI Reconstruction Using Many-Core Architectures

**DOI:** 10.1155/2011/473128

**Published:** 2011-09-14

**Authors:** Daehyun Kim, Joshua Trzasko, Mikhail Smelyanskiy, Clifton Haider, Pradeep Dubey, Armando Manduca

**Affiliations:** ^1^Parallel Computing Lab, Intel Corporation, 2200 Mission College Boulevard Santa Clara, CA 95054, USA; ^2^The Center for Advanced Imaging Research, Mayo Clinic, 200 First Street SW, Rochester, MN 55905, USA

## Abstract

Compressive sensing (CS) describes how sparse signals can be accurately reconstructed from many fewer samples than required by the Nyquist criterion. Since MRI scan duration is proportional to the number of acquired samples, CS has been gaining significant attention in MRI. However, the computationally intensive nature of CS reconstructions has precluded their use in routine clinical practice. In this work, we investigate how different throughput-oriented architectures can benefit one CS algorithm and what levels of acceleration are feasible on different modern platforms. We demonstrate that a CUDA-based code running on an NVIDIA Tesla C2050 GPU can reconstruct a 256 × 160 × 80 volume from an 8-channel acquisition in 19 seconds, which is in itself a significant improvement over the state of the art. We then show that Intel's Knights Ferry can perform the same 3D MRI reconstruction in only 12 seconds, bringing CS methods even closer to clinical viability.

## 1. Introduction and Motivation

Magnetic resonance imaging (MRI) is a noninvasive medical imaging modality commonly used to investigate soft tissues in the human body. Clinically, MRI is attractive as it offers flexibility, superior contrast resolution, and use of only nonionizing radiation. However, as the duration of a scan is directly proportional to the number of investigated spectral indices, obtaining high-resolution images under standard acquisition and reconstruction protocols can require a significant amount of time. Prolonged scan duration poses a number of challenges in a clinical setting. For example, during long examinations, patients often exhibit involuntary (e.g., respiration) and/or voluntary motion (e.g., active response to discomfort), both of which can impart spatial blurring that may compromise diagnosis. Also, high temporal resolution is often needed to accurately depict physiological processes. Under standard imaging protocols, spatial resolution must unfortunately be sacrificed to permit quicker scan termination or more frequent temporal updates.

Rather than executing a low spatial resolution exam, contemporary MRI protocols often acquire only a subset of the samples associated with a high-resolution exam and attempt to recover the image using alternative reconstruction methods such as homodyne detection [1] or compressive sensing (CS). CS theory asserts that the number of samples needed to form an accurate approximation of an image is largely determined by the image's underlying complexity [2, 3]. Thus, if there exists a means of transforming the image into a more efficient (i.e., sparse or compressible) representation, less time may actually be required to collect the data set needed to form the high-resolution image [4].

Background-suppressed contrast-enhanced MR angiography (CE-MRA) is a very natural clinical target for CS methods. As the diagnosis of many conditions like peripheral vascular disease are based on both vessel morphology and hemodynamics, high spatial and temporal resolution images are consequently needed. CS enables the acquisition of all of this information in a single exam. Although several authors (e.g., [5–8]) have successfully demonstrated the application of CS methods to CE-MRA, the computationally intensive nature of these applications has so far precluded their clinical viability (e.g., published CS reconstruction times for a single 3D volume (CE-MRA or not) are often on the order of hours [4, 7, 9–11]). As the results of a CE-MRA exam are often needed as soon as the acquisition completes (either for immediate clinical intervention or to guide additional scans), it is not practical to wait for the result of any currently implemented CS reconstructions. Instead, linear or other noniterative reconstructions that can be executed online (e.g., [12]) must be used even if they provide suboptimal results.

With the goal of reducing CS+MRI reconstruction times to clinically practical levels, several authors have recently considered the use of advanced hardware environments for their reconstruction implementations. Most of these techniques have focussed on the reconstruction of MRI data acquired using phased-array (i.e., multicoil) receivers, as this is the dominant acquisition strategy used in clinical practice. Chang and Ji [13, 14] considered a coil-by-coil approach to reconstructing phased-array MRI data. Although this strategy leads to natural task parallelization, with each element of a multicore processor independently handling the reconstruction on one coil image, they only demonstrated reconstruction times on the order of minutes, per 2D slice which is not clinically viable. Moreover, disjoint reconstruction of phased-array MRI data is well known to exhibit suboptimal performance when compared against joint reconstruction strategies like SENSE [15] and GRAPPA [16] and so, this strategy is of limited utility. Murphy et al. [17] later demonstrated that the SPIRiT reconstruction algorithm [18], which is a generalization of GRAPPA [16], can be significantly accelerated using graphics processors. They generated high-quality parallel image reconstructions in on the order of minutes per 3D volume, representing a significant advance towards clinical feasibility. More recently, Trzasko et al. [7, 8, 19] demonstrated CS reconstructions of time-resolved 3D CE-MRA images acquired using parallel imaging and a state-of-the-art Cartesian acquisition with a projection-reconstruction-like sampling (CAPR) strategy [12] also in a matter of only minutes per 3D volume using an advanced code implementation on a cluster system [20]. Their algorithm, which is essentially a generalization of SENSE [15] that employs auxiliary sparsity penalties and an efficient inexact quasi-Newton solver, was demonstrated to yield high quality reconstruction of 3D CE-MRA data acquired at acceleration rates upwards of 50x.

In this paper, we focus on Trzasko et al.'s CS+MRI reconstruction strategy for 3D CE-MRA and investigate the development, optimization, and performance analysis on several modern parallel architectures, including the latest quad- and six-core CPUs, NVIDIA GPUs, and Intel Many Integrated Core Architecture (Intel MIC). Our optimized implementation on a dual-socket six-core CPU is able to reconstruct a 256 × 160 × 80 volume of the neurovasculature from an 8-channel, 10x accelerated (i.e., 90% undersampled) data set within 35 seconds, which is more than a 3x improvement over other conventional implementations [8, 19]. Furthermore, we show that our CS implementation scales very well to the larger number of cores in today's throughput-oriented architectures. Our NVIDIA Tesla C2050 implementation reconstructs the same dataset in 19 seconds, while our Intel's Knights Ferry further reduces the reconstruction time to 12 seconds, which is considered clinically viable. Finally, our research simulator shows that the reconstruction can be done in 6 seconds on 128 cores, suggesting that many-core architectures are a promising platform for CS reconstruction.

## 2. Methods

### 2.1. Acquisition and Recovery of CE-MRA Images

Following [12], CAPR adopts a SENSE-type [15] parallel imaging strategy. As such, the targeted data acquisition process for one time frame can be modeled as

(1) $$\left[\begin{array}{c} g_{1}\\ g_{2}\\ \vdots \\ g_{C} \end{array}\right]=\left[\begin{array}{c} \Phi ℱ\Gamma_{1}\\ \Phi ℱ\Gamma_{2}\\ \vdots \\ \Phi ℱ\Gamma_{C} \end{array}\right]f+n,$$

where *f* is a discrete approximation of the underlying image of interest, Γ_*c*_ is the *c*th coil sensitivity function, *ℱ* is the 3D discrete Fourier transform (DFT) operator, Φ is a (binary) sampling operator that selects a prescribed subset of k-space values, *n* is complex additive white Gaussian noise (AWGN), and *g*_*c*_ is signal observed by the *c*th coil sensor. As described in [19], raw CAPR k-space data is background-subtracted and view-shared prior to execution of the CS reconstruction procedure. We let *h*_*c*_(*t*) denote the result after such preprocessing of *g*_*c*_.

It was demonstrated in [8, 19] that background-suppressed CE-MRA images acquired by systems of the form in (1) can be accurately recovered by (approximately) solving the following unconstrained optimization problem:

(2) $$\tilde{v}=\arg\underset{v}{\min\;}\;J\left(v\right),$$

where the cost functional

(3) $$J\left(v\right)=\alpha \sum_{n\in \eta }P\left(D_{n}v\right)+\overset{C}{\underset{c=1}{\sum }}{||\Phi ℱ\Gamma_{c}v-h_{c}||}_{2}^{2},$$

*D*_*n*_ is the finite spatial difference operator for some offset direction *n* (in the neighborhood *η*), and the penalty functional

(4) $$P\left(v\right)=\sum_{x\in \Omega }\rho \left(v\left(x\right)\right),$$

for some concave metric functional *ρ*(·) [21]. Following [7], the nonconvex Laplace functional

(5) $$\rho \left(\cdot \right)=1-\exp\;\left(\sigma^{-1}\left|\cdot \right|\right),\;\sigma \in \left[0,\infty \right)$$

is herein adopted. Although not considered here, the *ℓ*_1_-norm (*ρ*(·) = |·|) could also be used if so desired.

### 2.2. Numerical Optimization

In [7], an efficient inexact quasi-Newton algorithm was proposed for (approximately) solving (2) to reconstruct CAPR CE-MRA images. For completeness, this algorithm is briefly reviewed. Recall that complex quasi-Newton iterations [22] are typically of the form

(6) $$v_{i+1}=v_{i}-B^{-1}\left(v_{i}\right)L\left(v_{i}\right),$$

where the gradient of *J*(*v*) (taken with respect to $\bar{v}$ [23]) and *B*(·) is an approximation of the complex Hessian of *J*(*v*). The term “inexact” arises when Δ_*i*_ is only approximately determined, such as via truncated conjugate gradient (CG) iteration. More specifically, given (3),

(7) $$\begin{array}{cc} L\left(v_{i}\right) & =\alpha \sum_{n\in \eta }D_{n}^{*}\Lambda \left(D_{n}v_{i}\right)D_{n}v_{i}\\ & \;+\overset{C}{\underset{c=1}{\sum }}\Gamma_{c}^{*}{ℱ}^{*}\Phi^{*}\left(\Phi ℱ\Gamma_{c}v_{i}-k_{c}\left(t\right)\right), \end{array}$$

where the (*ϵ* > 0 smoothed) diagonal operator

(8) $$\Lambda {\left(D_{n}v_{i}\right)}_{\left(x,x\right)}=\frac{1}{2{\left|\left[D_{n}v_{i}\right]\left(x\right)\right|}_{\epsilon_{i}}}\cdot \frac{\partial \rho \left({\left|\left[D_{n}v_{i}\right]\left(x\right)\right|}_{\epsilon_{i}}\right)}{\partial {\left|\left[D_{n}v_{i}\right]\left(x\right)\right|}_{\epsilon_{i}}}.$$

In their work, Trzasko et al. [8, 19] adopted the following analytical linear Hessian approximation:

(9) $$B\left(v_{i}\right)=\frac{\alpha }{2}\sum_{n\in \eta }D_{n}^{*}\Lambda \left(D_{n}v_{i}\right)D_{n}+\overset{C}{\underset{c=1}{\sum }}\Gamma_{c}^{*}{ℱ}^{*}\Phi^{*}\Phi ℱ\Gamma_{c},$$

which can be considered a generalization of Vogel and Oman's “lagged diffusivity” model [24] for total variation (TV) denoising and deblurring. For improved convergence, decreasing continuation is also performed on the functional smoothing parameter, *ϵ* [25].

In [19], an efficient C++ implementation of the above algorithm employing the templated class framework described by Borisch et al. [20] and both the MPI (http://www-unix.mcs.anl.gov/mpi/) and OpenMP (http://www.openmp.org/) libraries was described and executed on an 8-node dedicated reconstruction cluster, where each node had two 3.4 GHz Intel Xeon processors and 16 GB memory. For a single 256 × 160 × 80 head volume reconstruction from 8-channel data and only 6 difference neighbors, reconstruction times of slightly less than 2 minutes were reported. Although these times represent a significant advancement over other existing works, they are still too long for routine clinical use.

## 3. Experiments

We used five datasets (two artificial and three clinical) to analyze the CS performance on three platforms: Intel CPUs, NVIDIA GPUs, and Intel MIC.

### 3.1. Experimental Data and Reconstruction Specifications

**Figure 1:**
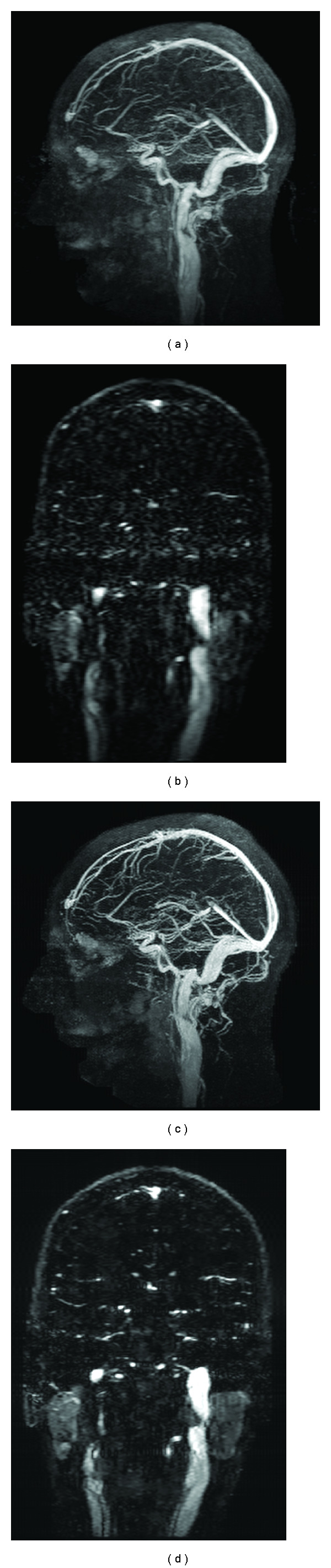
Sagittal maximum intensity projection (MIP) images (column 1) and coronal cross-section images (column 2) for test data set 5. (a-b) represent the current clinical reconstruction protocol result, and (c-d) represent the CS reconstruction. Note the relatively superior vascular conspicuity and parotid gland homogeneity in the CS reconstruction images.

Five datasets are used for our experiments, whose volume size and memory footprint are: (256 × 64 × 64, 224 MB), (256 × 160 × 32, 280 MB), (256 × 160 × 80, 700 MB), (256 × 160 × 84, 735 MB), and (256 × 160 × 88, 770 MB), in the order of dataset 1 to 5, respectively. Datasets 1 and 2 were artificially generated, Dataset 3 represents a noncontrast-enhanced brain, and Datasets 4 and 5 represent contrast-enhanced vasculature. All MRI data were acquired on a 3T GE Signa scanner (v.20) using an 8-channel head array using the CAPR acquisition sequence. Prior to reconstruction, view sharing was performed on datasets 3–5, and background reference subtraction on dataset 4 and 5 as described in [12]. For all experiments, 5 outer and 15 inner (CG) iterations were executed under *W* = 1 (corresponding to 26 finite difference neighbors). *ϵ*-continuation (0.1 reduction) was performed at each outer iteration.  Figure 1 shows the current clinical [12] and CS-type reconstruction [19] results for dataset 5. The CS reconstruction results for all versions optimized for different architectures discussed below were visually identical to that shown here. All architectures are compliant with IEEE single-precision floating-piont arithmetic standard [26].

### 3.2. Computing Architectures

**Intel Core i7 Processor**

The Intel Core i7 processor is an ×86-based multicore architecture which provides four/six cores (731 M/1.17 B transistors) on the same die. It features a superscalar out-of-order core supporting 2-way hyperthreading and 4-wide SIMD. Each core is backed by 32 KB L1 and 256 KB L2 caches, and all cores share an 8 MB/12 MB L3 cache. Quad and six-core CPUs provide 100 Gflops and 135 Gflops of peak single-precision computation respectively, as well 32 GB/s of peak memory bandwidth. To optimize CS on Core i7 processors, we took advantage of its SSE4 instructions using the Intel ICC auto-vectorizing compiler as well as hand-vectorization intrinsics. We parallelized the code with OpenMP and adopted a highly optimized FFT implementation from Intel's Math Kernel Library (*MKL*) 10.2.

**NVIDIA Tesla**

The NVIDIA Tesla C2050 [27, 28] (3 B transistors) provides 14 multiprocessors, each with 32 scalar processing units that share 128 KB of registers and a 64 KB on-chip memory. 32 scalar units are broken into two groups, where each group runs in lockstep. It features a hardware multithreading, which allows hundreds of thread contexts running concurrently to hide memory latency. All multiprocessors share a 768 KB L2 cache. The on-chip memory is software configurable, and it can be split into a 48 KB cache and a 16 KB shared memory space, or vice versa. Its peak single-precision computing performance is about 1.03 Tflops and its on-board GDDR memory provides up to 144 GB/s bandwidth. We used the CUDA [29] programming environment to implement CS on Tesla C2050. CUDA allows programmers to write a scalar program that is automatically organized into thread blocks to be run on multiprocessors. CUDA provides an open source FFT library (CUFFT 3.1 [30]) although more optimized FFT implementations such as Nukada and Matsouka's [31] have been published.

#### 3.2.1. Intel's Knights Ferry

Intel MIC is an Aubrey Isle- [32] based platform and Intel's Knights Ferry [33] is its first silicon implementation with 32 cores running at 1.2 GHz. It is an ×86-based many-core processor based on small in-order cores that combines the full programmability of today's general-purpose CPU architectures with the compute throughput and memory bandwidth capabilities of modern GPU architectures. Each core is a general-purpose processor, which has a scalar unit based on the Pentium processor design, as well as a vector unit that supports 16 32-bit float or integer operations per clock. It is equipped with two levels of cache: a low latency 32 KB L1 cache and a larger globally coherent total 8 MB L2 cache that is partitioned among the cores. It offers a peak throughput of 1.2 Tflops (single-precision). Because Intel MIC is based on ×86, it provides a natural extension to the conventional ×86 programming models. Thus, we could use similar data and thread level implementation as on Core i7 processors.

### 3.3. Assessment of Computational Burden

**Figure 2:**
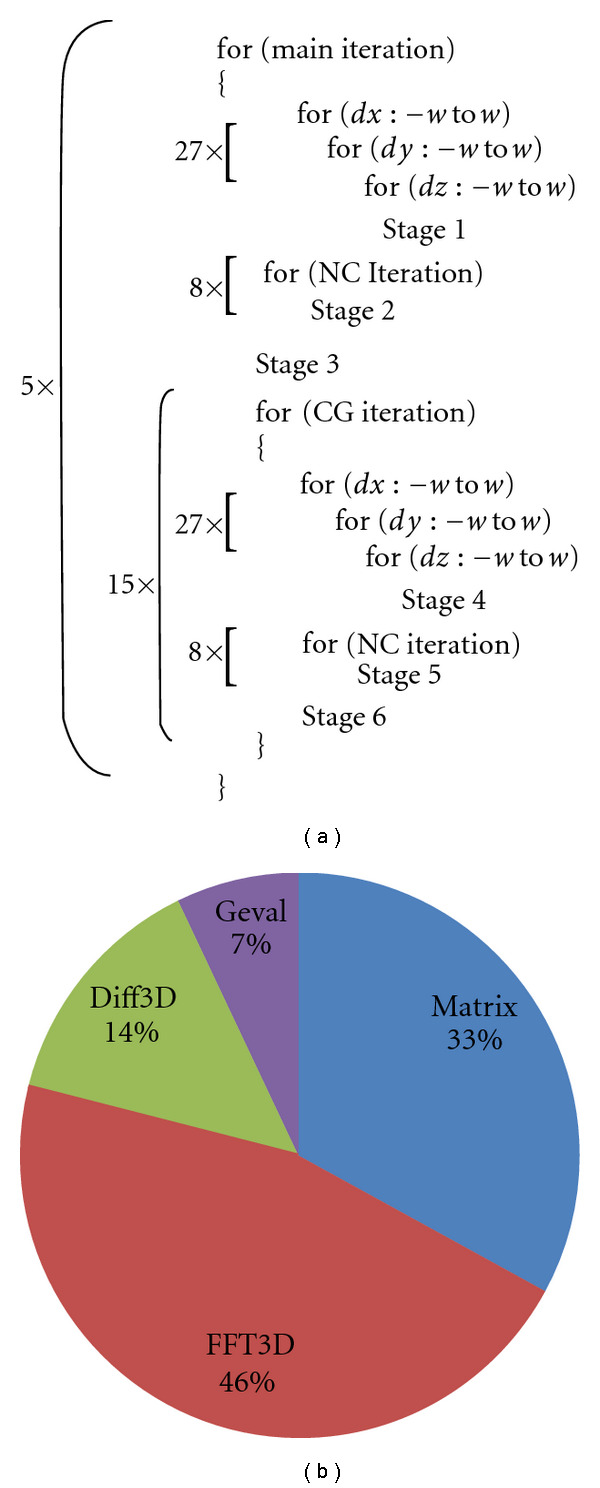
(a) CS implementation overview. (b) Execution time breakdown.

Figure 2(a) shows the overview of our CS implementation. The targeted CS reconstruction algorithm is composed of multiple iterations of 3D matrix arithmetics. We divide the reconstruction model outlined in (6) into six stages based on the loop structure (denoted as *Stage1*, *Stage2*, etc.). More specifically, *Stage1*, *Stage2*, and *Stage3* correspond with computation of the left and right terms of (7), respectively. Analogously, *Stage4*, *Stage5*, and *Stage6* correspond with computation of the left and right terms of (9), respectively. Each stage performs a series of matrix computations such as elementwise additions and 3D FFTs. The pie chart in Figure 2(b) shows the execution time breakdown of the key kernels. *FFT3D* (performed in *Stage2* and *Stage5*) is the most time-consuming and accounts for 46% of the total execution time. To achieve optimal performance, FFT requires architecture-specific optimization. Thus, we use the best FFT libraries available for each architecture. Simple elementwise matrix arithmetics (*Matrix*) are the second most time-consuming kernels. Because they stream large amount of data from/to the main memory, our optimizations focus on hiding latency and utilizing bandwidth efficiently. *Diff3D* (in *Stage1* and *Stage4*) calculates the differences from the original matrix to its shifted copy. Since the same data are used multiple times, we block the matrix to exploit data reuse in fast on-die memories. *GEval* (in *Stage1* and *Stage3*) performs transcendental operations such as division and exponentiation that are implicit within (8). On GPUs, we take advantage of fast math functions; however, the performance gain due to the faster math is marginal because *GEval* comprises only 7% of the total execution time.

## 4. Architecture-Aware Optimization

Architecture-aware optimization can improve performance significantly. Naive implementation often misses a large amount of performance potential. In order to realize maximum performance potential, we discuss a number of important architecture-aware optimizations for our CS implementation. Our optimization techniques are general, and thus can be applied to all three architectures (CPU, GPU, and Intel MIC). In particular, since a CPU and MIC share the same programming model, an optimized CPU code can be ported to Intel MIC without much modification. However, the CUDA programming model is quite different from a CPU's. It requires significant effort to port a CPU code to GPUs, and it may be easier to program it from scratch for GPUs. 

### 4.1. Vectorizing and Multithreading

We take advantage of modern parallel architectures through vectorizing and multithreading our CS implementation. The main data structures in CS are 3D matrices. Because each element of the matrix is computed independently in most kernels, we exploit the element-level parallelism. In other words, we can pack multiple elements into a vector computation and/or divide elements among multiple threads arbitrarily, without concern of data dependency.

**Figure 3:**
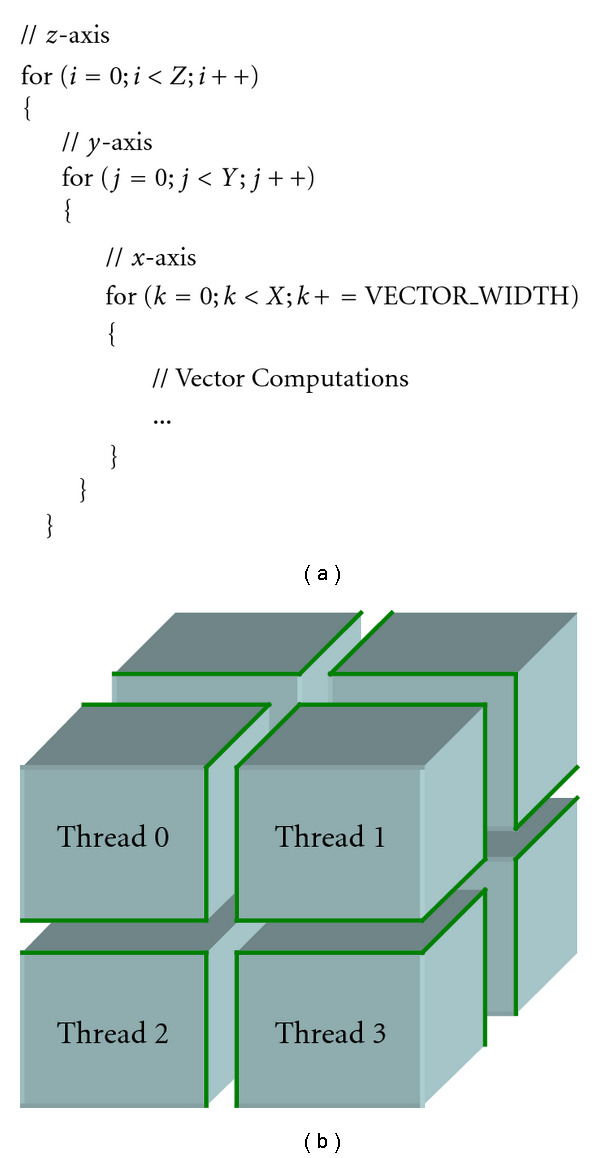
(a) Vectoring 3-nested loop. (b) Multithreading 3D matrix.

We start by vectorizing the inner-most loop of 3D matrix kernels. A kernel is usually is composed of three nested loops for each of the dimensions, *x*, *y*, and *z*, as shown in Figure 3(a). Because there is no data dependency between elements, it is possible to vectorize within any of the iterations. However, it is most efficient to vectorize the inner-most loop, since it exhibits sequential memory accesses along the *x* axis. Vectorization along the *y* or *z* axis requires gathering elements from nonsequential memory locations, resulting in poor performance. In addition, most loops in CS do not contain data-dependent control flow diversions (i.e., “if” statements), which helps maintain high vector efficiency.

Second, we perform 3D partitioning of the 3D matrix evenly among multiple threads, as shown in Figure 3(b). For each partition, the computation requirement is almost identical, and memory access patterns are very regular. Thus, our coarse-grain static partitioning provides good load balancing. Though fine-grain dynamic partitioning may provide better load balancing, it gives rise to interthread communication overhead. In dynamic partitioning, a partition that is assigned to a thread at one stage may be assigned to another thread at another stage, which incurs data communication from the initial thread to the next. In our static partitioning, a partition is always assigned to the same thread throughout multiple stages, thus interthread communication is not required.

It is not trivial to vectorize and multithread FFT due to its butterfly access patterns and bit-reversal element shuffling. However, this has been studied for decades and optimization techniques are well known. *FFT3D* optimization in our CS implementation uses techniques discussed in [34], details of which are out of scope of this paper.

### 4.2. Loop Fusion

**Figure 4:**
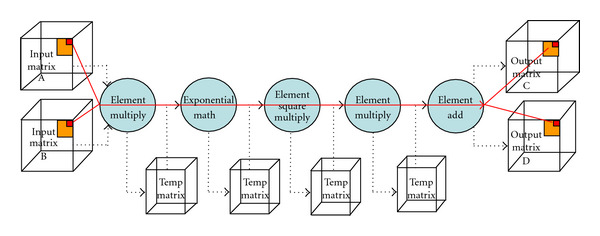
Example of loop fusion applied for *Stage3*.

Our CS implementation performs a series of simple elementwise matrix operations. For example, Figure 4 shows a high-level overview of *Stage3* which corresponds to assembly of the cost functional gradient defined in (7) following construction of its elements in *Stage1* and *Stage2*. This stage is composed of five computation substages for two input matrices, A and B, and two output matrices, C and D. More specifically, A contains the set of all intermediary data generated using *Diff3D* and B contains the set of all intermediary data generated using *FFT3D*. C then corresponds to the weighting matrix defined in (8), whereas D is the entire composite variable in (7). One possible implementation is to execute each computation stage entirely before proceeding to the next state, as illustrated with the dotted arrows in the figure. For example, we first multiply matrices A and B, then we perform an exponentiation of the result, and so forth. While this approach is easy to implement, its memory behavior is inefficient. Because each stage sweeps through the entire 3D matrix and the size of the matrix is usually larger than the last level cache size, temporary matrices between stages cannot be retained within the cache, which results in cache thrashing, memory traffic increase, and, therefore, overall performance degradation. A better implementation is to block the computation so that its temporary data is kept within the cache. Main memory accesses will occur only at the beginning to read the input matrix and at the end to write the output matrix. We optimize even further to process the entire computation at the element level as shown in the solid arrow in the figure. We read an element from each of matrix A and matrix B, perform the five computations, and write the result to matrix C and D. Then, we move onto the next element, and so on. This optimization is called loop fusion, because it fuses multiple small loops of individual computations into one big loop of a combined computation. Because it handles one element at a time, data can be kept in the registers, which eliminates the need for the temporary matrix and, therefore, removes intermediate memory loads/stores completely. Also, because a fused loop performs more computation before it accesses the next element, it has more time to hide memory latency through data prefetches.

### 4.3. Cache Blocking through Data Partitioning

**Figure 5:**
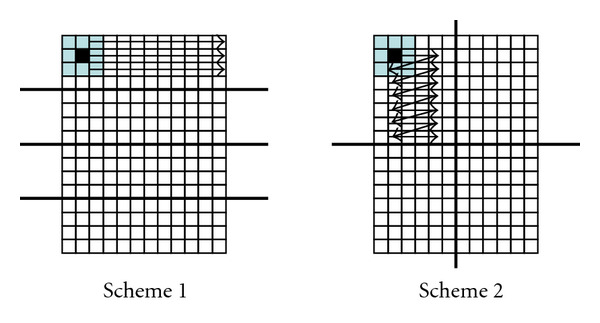
Cache blocking through data partitioning.

Most matrix operations in CS read/write only one element from an input matrix to an output matrix. Once an element is processed, the same element is not accessed again. However, *Diff3D* requires 27 neighbor elements to compute an output element. In other words, an input element is reused 27 times for 27 different output elements. To capture the data reuse, a cache-aware partitioning is required. For brevity, we explain our optimization with a 2D matrix shown in Figure 5. To compute an output, it requires 9 surrounding inputs. Scheme  1 is a cache-ignorant partitioning. It accesses elements from the beginning to the end of the current row before accessing elements in the the next row. It fails to capture data reuse if the matrix is too large. Initially, it caches the first 3 × 3 inputs to calculate the first output. But when it reaches the end of the row, the first 3 × 3 inputs are likely to be evicted from the cache if the number of elements touched during the row traversal exceeds the cache size. As a result, 3 × 3 inputs are reloaded from memory to calculate the first output of the next row even though 2 × 3 of these inputs have been already read before. Scheme  2 solves this problem by a cache-aware partitioning. It partitions the matrix in the middle of the row. Instead of moving to the end of a row, it stops at the end of the partition and moves down to the next row. It can reuse the inputs from the previous row before they are replaced from the cache. When we divide a matrix into four partitions, scheme  2 will show better cache behavior than scheme  1. We extend the same cache-aware data partitioning technique to a 3D matrix to implement *Diff3D*.

### 4.4. Cache Line Padding for 3D Matrix

**Figure 6:**
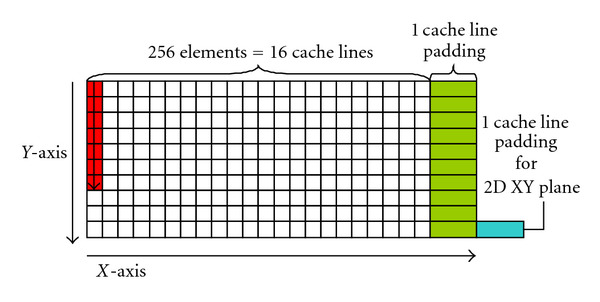
Cache line padding for 3D matrix.

Though the main data structure in CS is a 3D matrix, the main memory access pattern is simple streaming that accesses data from the first to the last sequentially. However, *Diff3D* and *FFT3D* also access data nonsequentially along the *y* and *z* axis. Memory accesses with a large power-of-two stride show poor cache behavior due to cache conflicts. For example, this can occur when multiple data elements from adjacent matrix rows map to the same cache line. As the result, access to the second element results in the eviction of the first element from the cache. To solve the problem, we pad the matrix as shown in Figure 6. Each row along the *X*-axis is padded with one empty cache line at the end. Without padding, accesses along the *Y*-axis have stride of 16 cache lines (power of two). We break the power-of-two stride by adding one extra cache line per row, which will reduce cache conflict misses. Note that, in addition to the padding at the end of row along the *X*-axis, we may also need to add another padding at the end of each *XY* 2D plane, if the size of *Y* dimension is also power of two.

### 4.5. Last-Level Cache Blocking for 3D FFT

**Figure 7:**
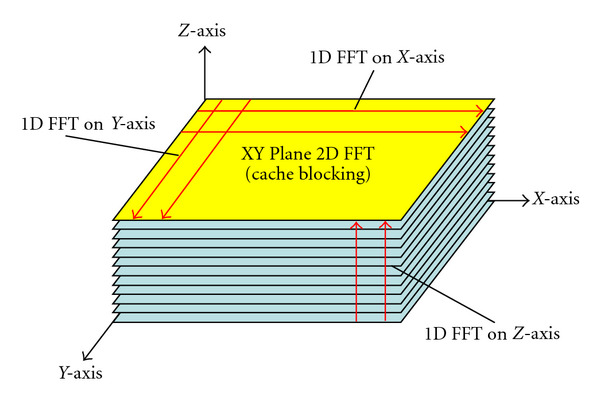
Last-level cache blocking for 3D FFT.

3D FFT can be computed as multiple 1D FFTs along the *X*-, *Y*- and *Z*-axis. For a 256 × 160 × 80 matrix as our reference dataset, we can first perform 12800 256-point 1D FFTs along the *X*-axis, followed by 20480 160-point 1D FFTs along the *Y*-axis, and finally 40960 80-point 1D FFTs along the *Z*-axis. However, performing 1D FFTs, one axis at a time is not cache efficient, because it requires sweeping the entire matrix, which incurs a lot of cache misses due to the fact that the matrix does not fit into last-level cache. Instead, we perform 2D FFTs for each 2D *XY* plane, then we perform 1D FFTs for the *z* axis, shown in Figure 7. For a given *z* axis value, we preload the corresponding *XY* plane to the cache, perform 1D FFTs along the *X*-axis then the *y* axis (a 2D FFT for the entire *XY* plane), and then store the resulting *XY* plane to the memory. Because last-level caches are usually larger than a single *XY* plane (320 KB for the reference dataset), our *XY* plane cache blocking is very effective in reducing memory bandwidth requirements. Note that larger last-level caches in Intel Core i7 processor and Intel's Knights Ferry than Tesla C2050 are beneficial for the 2D cache blocking. As each thread/core works on a different *XY* plane, multiple 2D blocks should be kept in the cache. In addition, as the size of datasets increases, the corresponding 2D blocks also get larger. Therefore, to achieve good system-level and dataset-level scalability, large last-level caches are critical.

### 4.6. Synchronization: Barrier and Reduction

CS is composed of a large number of parallel stages separated by global barriers (routines that synchronize threads in a parallel system). When threads finish computation in the parallel region, they synchronize on a barrier before proceeding to the next parallel region. Efficient barrier synchronization is paramount for high scalability. On a system with a small number of cores such as Intel Core i7 processor, barrier overhead is only *∼*2% of total execution time. But as we increase the number of threads (i.e., 128 threads on Intel's Knights Ferry), we observe up to *∼*10% overhead with our hand-optimized barrier implementation. Without the optimized barrier, the synchronization overhead would be too large, therefore resulting in poor performance. On GPUs, the barrier overhead is even worse. We implement a global barrier by launching a new kernel, that is, synchronizing with CPU, which costs one kernel launch overhead at minimum. While there exist faster barrier implementations that run entirely on GPU, they require nonstandard memory consistency model assumption.

Another synchronization primitive used in CS is reduction. In *Stage3* and *Stage6*, it reduces a 3D matrix to one scalar value. To implement this reduction, we used a software privatization technique. Each thread performs local reduction into its private scalar variable. After all threads are done, the global reduction is performed, which aggregates all local values. To implement the global reduction, we use an atomic memory operation. While atomics are generally slow on modern parallel architectures, their overhead in our CS implementation is small, due to the small fraction of time spent in the global reduction.

## 5. Results

We compare performance of our CS implementation on three modern parallel architectures and provide in-depth performance analysis from a computer architecture perspective.

### 5.1. Impact of Performance Optimization

**Figure 8:**
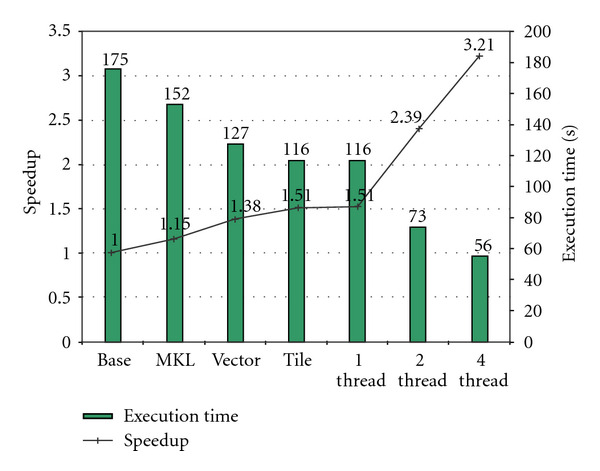
Impact of optimization on a quad-core CPU.

We applied the various optimizations discussed in Section 4 to our CS implementation. We demonstrate the impact of each individual optimization on overall performance.  Figure 8 shows the performance improvement of our CS implementation first as a single-threaded program on an Intel Core i7 processor as we incrementally applied our optimizations and subsequently as a multithreaded program. The vertical bar represents the execution time in seconds for each optimization step, and the line shows the corresponding relative speedup over the baseline, *Base*, which is the original single-threaded implementation of the algorithm compiled with the highest level of optimization, including autovectorization, function in-lining, and interprocedural optimization. As our first optimization, we replaced the FFTW [35] used in the original implementation with the faster Intel MKL. This results in a 1.15x speedup as represented by the second bar *MKL*. Second, we hand-vectorize the codes that can not be autovectorized by the compiler. Hand-vectorization provides an additional 1.19x speedup (*Vector*). Third, we apply cache blocking to exploit data reuse in the *Diff3D* kernel, which shows another 1.10x speedup (*Tile*). Through these three single-thread optimizations, we achieve an overall 1.51x speedup over the baseline implementation. To take advantage of multiple cores/threads, we parallelize the application. For *FFT3D*, we use the parallel implementation of the MKL library, and for the other kernels, we hand-parallelize using the OpenMP library. Parallelization achieves another 1.58x speedup on two cores over the single-core baseline and a 2.14x speedup on four cores. Overall, by combining the single-thread optimization and the multithread parallelization, we achieve a 3.21x performance improvement from the baseline implementation, which reduces the total execution time from 175 seconds (*Base*) to 56 seconds (*4 Cores*).

### 5.2. Performance Comparison: CPU, GPU, and Intel MIC

**Figure 9:**
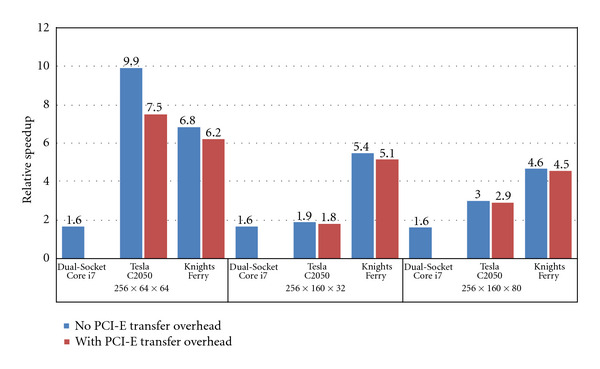
Performance comparison between Dual-Socket Core i7, Tesla C2050, and Knights Ferry with respect to Quad-Core Core i7.

Figure 9 compares CS performance on three architectures: Intel dual-socket six-core Core i7 processor (Intel Xeon processor X5670 at 2.93 GHz), NVIDIA Tesla C2050 (at 1.15 GHz attached to Intel Core i7 processor 960 at 3.2 GHz), and Intel's Knights Ferry (at 1.2 GHz attached to Intel Core i7 processor 960 at 3.2 GHz). We normalize the speedups with respect to the optimized quad-core Core i7 processor (Intel Core i7 processor 975 at 3.33 GHz) implementation (56 second runtime) from the previous section and show them only for dataset 1, 2, and 3. Since their dimensions are similar, the performance results for datasets 4 and 5 are correspondingly very similar to those for dataset 3 in terms of both execution time and relative performance across different architectures. Thus, for the sake of brevity, only the results for dataset 3 are shown in Figure 9. For Tesla C2050 and Knights Ferry, we show two speedup bars: one without data transfer overhead from the CPU host and the other with the overhead. The data transfer overhead results in small performance degradation, because CS spends significant time performing computation, and can hide most of the data transfer time.

The dual-socket six-core Core i7 processor (total 12 cores) performs about 1.6x faster (35 s) than the quad-core CPU (56 s), thanks to the increased core count and memory bandwidth. The Tesla C2050 GPU platform is 2.9x faster than the quad-core CPU for dataset 3. However, its performance exhibits large variance across datasets. Tesla C2050 shows about 7.5x speedup for dataset 1 but shows only 1.8x speedup for dataset 2. For dataset 3 (actual clinical data), Knights Ferry achieves 4.5x speedup over the quad-core CPU, which is about 1.57x faster than Tesla C2050. Knights Ferry allows reconstructing the real anatomical dataset within a clinically feasible 12 seconds, which is a significant improvement over existing CS implementations.

Note that Core i7 processor and Knights Ferry are more efficient than Tesla C2050 in terms of resource utilization. Though Tesla C2050 has about *∼*4x peak flops and *∼*3x peak bandwidth than Core i7 processor, it only provides *∼*2x performance. Also, while Knights Ferry delivers *∼*10%± flops/bandwidth of Tesla C2050, Knights Ferry shows about 55% speedup over Tesla C2050. Finally, Tesla C2050's big performance variance across datasets is due to FFT optimization. We believe that CUFFT 3.1 is specially optimized for small power of two datasets like dataset 1. Thus, its FFT performance on dataset 1 is significantly better than on dataset 2 and 3.

## 6. Discussion

To elucidate the apparent impact architecture choice has on CS performance, we now provide an in-depth discussion on the performance of kernel-level operations. We then discuss the parallel scalability of CS performance to address future CMP systems.

### 6.1. In-Depth Performance Analysis

We provide further insights into the achieved performance by breaking down the entire application into small microkernels and analyzing the microkernels individually. We focus on the Intel quad-core Core i7 processor and Intel's Knights Ferry due to the lack of performance analysis tools in NVIDIA's CUDA environments.  Table 1 shows the summary of our analysis. There are two columns for each kernel. The first column shows the fraction of execution time spent in the kernel inside the sequential code. The second column shows the speedup achieved by Knights Ferry over Core i7 processor on the kernel. Dataset 3 (single-precision complex 256 × 160 × 80) is used for the analysis.

FFT3D is the most important kernel occupying 46% of the total execution time. For the reference dataset, the Intel MKL library achieves *∼*30 Gflops on Core i7 processor and our in-house FFT library achieves *∼*175 Gflops on Knights Ferry, which are the best performances that can be achieved on both architectures today. Therefore, for *FFT3D*, we estimated *∼*6x speedup of Knights Ferry over Core i7 processor and actually obtained *∼*4x speedup, which indicates that we might improve Knights Ferry performance further.

*Diff3D* performs a 2-point convolution. It subtracts the original matrix and its shifted matrix by *dx*, *dy*, and *dz*: *out = In-In_Shifted( dx*, *dy*, *dz*). Because the computation is a simple subtraction and the data size is large (*∼*200 MB), it is bandwidth bound. Considering the memory bandwidth of Core i7 processor and Knights Ferry, we expected *∼*4x speedup of Knights Ferry over Core i7 processor. In actual implementation, we achieved *∼*6x speedup, which indicates that Core i7 processor may have room to improve.

*GEval* involves transcendental operations. In our hand-optimized microbenchmark, exponentiation takes *∼*37 cycles/element in Core i7 processor and *∼*0.88 cycles/element in Knights Ferry, and square-root computation takes *∼*4.5 cycles/element in Core i7 processor and *∼*0.56 cycles/element in Knights Ferry. Based on the microbenchmark performance, we estimated *∼*8x speedup of Knights Ferry over Core i7 processor, and its actual performance (*∼*7x) is close to our estimation.

*Matrix* computes simple element-wise arithmetics. In many cases, it is bandwidth bounded, because computations are simple addition, subtraction, or multiplication. However, we also optimize it by fusing multiple computations together to exploit register and cache blocking. Considering the peak memory bandwidth on both architectures, Knights Ferry is expected to be *∼*4x faster than Core i7 processor. Considering the peak floating point performance, Knights Ferry is expected to be *∼*12x faster than Core i7 processor. In reality, we achieved *∼*4x speedup of Knights Ferry over Core i7 processor, which indicates that the kernel is currently bandwidth-bound on both architectures.

### 6.2. Future CMP Scalability

**Figure 10:**
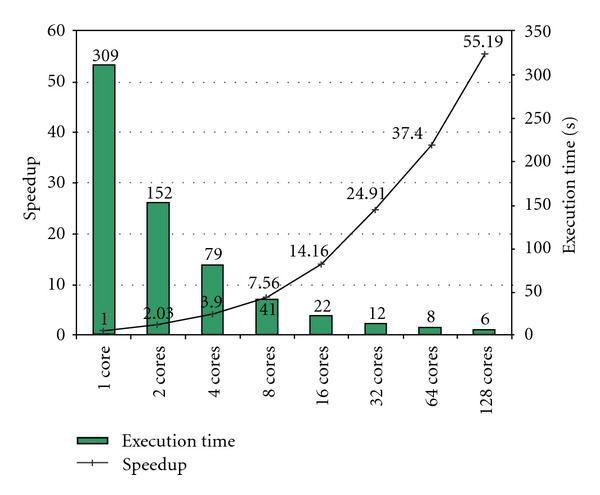
Performance scalability of future many-core implementations.

 Chip multiprocessors (CMPs) provide applications with an opportunity to achieve much higher performance, as the number of cores continues to grow over time in accordance with Moore's law. For CMPs to live up to their promise, it is important that as the number of cores continues to grow the performance of the application running on CMPs also increases commensurately. To gain insights into how the CS algorithm will scale on future many-core architectures, we modeled a feasible but hypothetical future CMP processor (running at 1 GHz with 256 KB/core cache) on our cycle-accurate research simulator and Figure 10 shows its CS performance scalability. We observe that CS scales well as we increase the number of cores. In particular, our 64-core configuration achieves 37x speedup over a single-core configuration, which is almost 60% parallel efficiency. Moving further, our 128-core configuration achieves 55x speedup over single-core configuration which is little less than 50% efficiency.  Table 2 compiles the predicted 128-core acceleration together with the previously discussed results for Intel Core i7 processor, NVIDIA Tesla C2050, and Intel's Knights Ferry. Although a 256 × 160 × 80 data volume may be considered large by numerical computing standards, it is relatively small by modern clinical standards and volumes many times larger are routinely encountered in practice. Moreover, many contemporary acquisition trends are migrating from single-phase to time-resolved paradigms, where a 3D “movie” of dynamic anatomy and physiology (e.g., of contrast flowing into and out of vessels) is created. Thus, the scalability of CMPs is paramount to seeing CS-type and other nonlinear reconstruction methods become practical for such imaging scenarios. Overall, existing and future many-core architectures are very promising platforms for accelerating the CS reconstruction algorithm to make it clinically viable. 

## 7. Conclusion

In this work, we have shown that advanced computing architectures can facilitate significant improvements in the performance of CS MRI reconstructions and particularly that optimized use of modern many-core architectures can significantly diminish the computational barrier associated with this class of techniques. This suggests that as many-core architectures continue to evolve, CS methods can be employed in routine clinical MRI practice. Although CE-MRA was targeted in this work, the implication of the results apply to many other MRI applications as well as other areas in medical imaging such as dose reduction in computed tomography.

## Figures and Tables

**Table 1:** Performance analysis.

Kernel	Execution time breakdown	Speedup of Knights Ferry/Core i7
FFT3D	46%	*∼*4x
Diff3D	14%	*∼*6x
GEval	7%	*∼*7x
Matrix	33%	*∼*4x

Overall	100%	4.6x

**Table 2:** Summary of performance across different hardware.

	Execution time	Speedup
Core i7 processor 1 Core, Not Optimized	175 s	1.0x
Core i7 processor 1 Core, Optimized	116 s	1.5x
Literature Best [19]	*∼*100 s	1.7x
Core i7 processor 4 Cores	56 s	3.1x
Core i7 processor Dual-Socket 12 Cores	35 s	5.0x
NVIDIA Tesla C2050	19 s	9.2x
Intel's Knights Ferry (32 Cores)	12 s	14.5x
Research CMP Simulation (128 Cores)	6 s	29.1x
